# Supplementary material for: Geometry of braided DNA dictates supercoiling partitioning
Source: Nat Commun. 2026 Sep 8;17:10600. doi: 10.1038/s41467-026-77356-9 (PMC13646288; doi:10.1038/s41467-026-77356-9)
Supplement: Supplementary file 2 [file 41467_2026_77356_MOESM2_ESM.pdf]

nature portfolio

Corresponding author(s): Michelle D. Wang

Last updated by author(s): Aug 1, 2026

Reporting Summary

Nature Portfolio wishes to improve the reproducibility of the work that we publish. This form provides structure for consistency and transparency in reporting. For further information on Nature Portfolio policies, see our Editorial Policies and the Editorial Policy Checklist.

Statistics

For all statistical analyses, confirm that the following items are present in the figure legend, table legend, main text, or Methods section.

n/a	Confirmed	
☐	☒	The exact sample size (*n*) for each experimental group/condition, given as a discrete number and unit of measurement
☐	☒	A statement on whether measurements were taken from distinct samples or whether the same sample was measured repeatedly
☒	☐	The statistical test(s) used AND whether they are one- or two-sided<br>*Only common tests should be described solely by name; describe more complex techniques in the Methods section.*
☒	☐	A description of all covariates tested
☒	☐	A description of any assumptions or corrections, such as tests of normality and adjustment for multiple comparisons
☐	☒	A full description of the statistical parameters including central tendency (e.g. means) or other basic estimates (e.g. regression coefficient) AND variation (e.g. standard deviation) or associated estimates of uncertainty (e.g. confidence intervals)
☒	☐	For null hypothesis testing, the test statistic (e.g. *F*, *t*, *r*) with confidence intervals, effect sizes, degrees of freedom and *P* value noted<br>*Give P values as exact values whenever suitable.*
☒	☐	For Bayesian analysis, information on the choice of priors and Markov chain Monte Carlo settings
☒	☐	For hierarchical and complex designs, identification of the appropriate level for tests and full reporting of outcomes
☒	☐	Estimates of effect sizes (e.g. Cohen's *d*, Pearson's *r*), indicating how they were calculated

Our web collection on statistics for biologists contains articles on many of the points above.

Software and code

Policy information about availability of computer code

Data collection	AOT software: LabVIEW 8.5 ; MC Simulation Software: MATLAB 2024b
Data analysis	AOT experiment analysis: MATLAB R2020a trace analysis software<br>The data analysis script and the simulation code can be accessed from Zenodo: https://doi.org/10.5281/zenodo.21752515.

For manuscripts utilizing custom algorithms or software that are central to the research but not yet described in published literature, software must be made available to editors and reviewers. We strongly encourage code deposition in a community repository (e.g. GitHub). See the Nature Portfolio guidelines for submitting code & software for further information.

Data

Policy information about availability of data

All manuscripts must include a data availability statement. This statement should provide the following information, where applicable:

- Accession codes, unique identifiers, or web links for publicly available datasets
- A description of any restrictions on data availability
- For clinical datasets or third party data, please ensure that the statement adheres to our policy

All data generated in this study have been deposited in the Source Data folder in Zenodo: https://doi.org/10.5281/zenodo.21752515.

Research involving human participants, their data, or biological material

Policy information about studies with human participants or human data. See also policy information about sex, gender (identity/presentation), and sexual orientation and race, ethnicity and racism.

Reporting on sex and gender	NA
Reporting on race, ethnicity, or other socially relevant groupings	NA
Population characteristics	NA
Recruitment	NA
Ethics oversight	NA

Note that full information on the approval of the study protocol must also be provided in the manuscript.

Field-specific reporting

Please select the one below that is the best fit for your research. If you are not sure, read the appropriate sections before making your selection.

☒ Life sciences ☐ Behavioural & social sciences ☐ Ecological, evolutionary & environmental sciences

For a reference copy of the document with all sections, see nature.com/documents/nr-reporting-summary-flat.pdf

Life sciences study design

All studies must disclose on these points even when the disclosure is negative.

Sample size	No formal sample size calculation was performed. Each data point corresponds to a unique DNA molecule. The sample size was based on prior experience with single-molecule measurements and established practices in the field. The number of replicates for each experimental condition was between N = 5 and 20. Detailed statistical information, including the number of traces, mean values, and standard deviation (SD), is provided in the manuscript text, figures, and figure legends.
Data exclusions	Traces are excluded from further analysis for the following reasons: instrument drift during the collection of the trace, DNA tether breakage or loss of torsional constraint, multiple molecule tethering events.
Replication	Traces were collected from multiple sample chambers. Traces collected from different sample chambers gave reproducible results. Each sample chamber can yield about 1-10 traces.
Randomization	Randomization was not relevant to this study; all experiments were carried out using the same experimental procedures.
Blinding	Blinding was not relevant to this study. All data collection and analysis were performed using standardized procedures that were applied uniformly across all samples and experimental conditions. All data collection and analysis were based on objective, quantitative measurements obtained from single-molecule optical tweezers experiments.

Reporting for specific materials, systems and methods

We require information from authors about some types of materials, experimental systems and methods used in many studies. Here, indicate whether each material, system or method listed is relevant to your study. If you are not sure if a list item applies to your research, read the appropriate section before selecting a response.

Materials & experimental systems

n/a	Involved in the study	
☐	☒	Antibodies
☒	☐	Eukaryotic cell lines
☒	☐	Palaeontology and archaeology
☒	☐	Animals and other organisms
☒	☐	Clinical data
☒	☐	Dual use research of concern
☒	☐	Plants

Methods

n/a	Involved in the study	
☒	☐	ChIP-seq
☒	☐	Flow cytometry
☒	☐	MRI-based neuroimaging

Antibodies

Antibodies used	Anti-digoxigenin, Roche, 11333089001, Lot# 5108900.
Validation	Anti-digoxigenin was used to tether DNA to a quartz cylinder. It was validated by the observation of DNA labeled with digoxigenin tethered to the cylinder.

Plants

Seed stocks	NA
Novel plant genotypes	NA
Authentication	NA
