# Supplementary material for: Geometry of braided DNA dictates supercoiling partitioning
Source: Nat Commun. 2026 Sep 8;17:10600. doi: 10.1038/s41467-026-77356-9 (PMC13646288; doi:10.1038/s41467-026-77356-9)
Supplement: Supplementary file 3 [file 41467_2026_77356_MOESM3_ESM.pdf]

nature portfolio

Peer Review File

Geometry of Braided DNA Dictates Supercoiling Partitioning

Corresponding Author: Professor Michelle Wang

This file contains all reviewer reports in order by version, followed by all author rebuttals in order by version.

Version 0:

Reviewer comments:

Reviewer #1

(Remarks to the Author)

Review of Geometry of Braided DNA Dictates Supercoiling Partitioning

In the report by Hong et al. entitled 'Geometry of Braided DNA Dictates Supercoiling Partitioning' the authors describe a sophisticated optical trapping technique that permits direct measurement of torque during the braiding of two double-stranded DNA molecules. DNA braids are employed to model the behavior of DNA catenanes that form in vivo as replication intermediates. Extensive literature from the past half century has characterized the topological properties of DNA during replication and the propensity of DNA to form braid-like structures as a consequence of torsional strain that accumulates ahead of a progressing replication fork. The physics of DNA braiding and the impact of DNA braid chirality on the catalytic activities of replication-associated enzymes has been reported over the past twenty years.

The main advance of the present work is the well-controlled braiding geometry that the authors achieve using a clever molecular biology approach to modify the DNA molecules used in the study. The approach permits them to generate O-braides, where the DNA ends are relatively close, on the order of one persistence length of dsDNA (~150nm), V-braids where one set of ends are close and the others are much further apart, and U-braids where both sets of ends are far apart. The authors relate the O-braid separation to the spacing that might be expected for DNAs associated with the actively progression replication machinery - however, this distance is likely to be much smaller than the end separation in the O-braid geometry.

The authors report a detailed biophysical characterization of the braiding behavior of each type of braid, and in particular the specific optical trapping technique developed years ago in the author's lab allows direct measurement of the braiding density - torque relationship. Previous single molecule studies of DNA braids by multiple research groups arrived at somewhat similar conclusions to the present work, but relied on simulations and geometric models to extract information about the torque response of DNA braids.

There is no doubt that the present work provides a higher resolution view and detailed torque response characterization of DNA braids with different geometries - showing that when two of the DNA ends are far apart there is a significant torque overshoot that occurs during the initial formation of the first DNA braid crossing. In other words, the energetic penalty for collapsing two DNA molecules with ends that are anchored far from each other is much larger than when the ends are close. This single molecule response was reported previously for less controlled DNA braiding systems, but the specific torque characterization in this study is an advance from the prior work.

While the authors cite all of the appropriate literature in this current manuscript, they do not place the current work sufficiently into the context of the previous single molecule studies so as to highlight how their results represent a significant advancement. Moreover, while the authors speculate that braid geometry may represent a novel mode of regulation of DNA structures in vivo, they do not perform any experiments that directly support or test this interesting hypothesis. Thus, the work by Hong et al. is a more biophysically focussed study whose biological relevance remains to be appropriately explored. For this reason, it is not clear that the well-described study of torque-dependence of DNA braiding on different geometries will be of sufficient interest to a broad readership to justify its publication in Nature Communications.

(Remarks on code availability)

Reviewer #2

(Remarks to the Author)
In this manuscript, Hong et al. measure the torsional response of braided DNA. The measurements in this study provide a significant advance beyond prior work on this topic, by using more defined DNA substrates than were previously employed and by collecting detailed datasets with direct torque measurements. The measurements are relevant to understanding the behavior of intertwined DNA duplexes in biology, including during replication where supercoiling can partition in front and behind the fork (as the authors emphasize and as Wang and coworkers previously investigated in elegant working using chromatinized DNA). The measurements are also of general interest in understanding the physics of intertwined polymers, and provide valuable data for comparison with theoretical treatments. The questions pursued in this study are thus important, and the measurements are a novel and substantial contribution. However, the authors should re-examine their analysis and conclusions before the study can be published.

In Figure 5b, supported by Table S2, the authors model a forked DNA molecule under conditions where torque(n) measurements of a braid produce a torque “overshoot”. They assume that no supercoiling can partition into the braid at torques below the overshoot. This will not in general be true for an equilibrium treatment of a braid in series with a single torsionally constrained DNA molecule. The torque overshoot is measured in an experiment where torque(n) is obtained in the fixed-n ensemble, and the result is a nonmonotonic function where the same torque is reached at multiple values of n. To model the forked system, a statistical mechanical treatment is needed that considers all possible values of the fluctuating braid catenation number (n) while fixing the total supercoiling across the forked DNA including the single-DNA section. In the limit of a long (and hence torsionally soft) appended single-DNA section, the behavior of the braid section will approach the fixed-torque ensemble — where the distribution P(n) at a given fixed torque will be bimodal for a range of torques below the overshoot torque, as implied by the shape of the nonmonotonic torque(n) measured above. See previous work on torque “overshoots” in plectonemic DNA, B-Z transitions, etc. The above assumes an equilibrium treatment, which I think was the implied intent of the authors; if they wish to propose a *kinetic* barrier to braid initiation then this should be explicitly described, motivated, and justified.

Related to the above, when investigating partitioning across the fork, the authors should discuss the relationships of the regimes they consider to plausible biological conditions during replication. At a replication fork, it is not immediately clear how the DNA separation at *both* ends of the braid can be large (as modeled in 5b), given the relatively small size of the replication machinery. For all modeled conditions (Fig 5ab, Fig S5), the authors consider a regime of moderate tension (2 pN) and moderate supercoiling where buckling does not occur. In the cell, lower tensions where buckling occurs are likely to be an important regime. (In fact the DNA is drawn as buckled in the fork cartoon provided by the authors in 5b, a picture that is not representative of the conditions modeled in partitioning calculations.) The partitioning between the single-DNA and the braid can be quite different in a regime where the DNA buckles. The authors may consider expanding the regimes investigated.

Experimentally, the authors should show torque-twist curves taken in both directions (unwinding/rewinding) in order to clearly establish whether the overshoot features are representative of the equilibrium torque-twist relationship.

Another experimental point that should be clarified is the topology of the V substrate. The construction method implies that one of the arms will be torsionally constrained, and the construct is described that way in the Methods. In the main text it is stated that both arms are nicked before the measurement, but it should be explained how this is ensured — or whether and how tethers are selected for serendipitous cases where both arms are unconstrained.

In general the authors should be careful to clearly and accurately describe the steps and assumptions in the derivation of theoretical expressions, even when they closely follow previous work. E.g. the supplement implies that eq. 5 follows directly from eq. 2 via a Maxwell relation, which is not the case. A longer discussion of the limitations of the geometrical model would be appropriate, including plotting eq. 1-2 and 4-5 overlaid on plots of extension and torque vs supercoiling.

Limitations of the experiment should also be discussed at more length — while substrates are more well-defined than previous work, the end separation is still not controlled for the open ends of V and U substrates; single-DNA results for comparison with braids rely on past literature rather than side-by-side comparisons under the same conditions; forked DNA is modeled rather than directly experimentally measured; and limited torque resolution seems to preclude detecting smaller overshoots or clearly characterizing the shape of the torque response during the first half turn.

Minor: on page 9, “the torque overshoot and the torque gap are proportional…” — I think the authors mean “hat tip” and not “torque gap”.

The authors may miss an opportunity for detailed comparisons with more sophisticated theory; it is reasonable for this to be left for future work, but will be important for the authors to provide accessible data for others to make use of these important new experimental results.

(Remarks on code availability)

Reviewer #3

(Remarks to the Author)
Hong and co-authors employ the angular optical trap (AOT) to measure the torque associated with “braiding” or wrapping to double stranded DNA molecules around each other, akin to precatenane formation occulting in daughter DNA molecules following replication. The authors develop different DNA constructs to tether the optically trapped quartz cylinder to the surface in three different geometries: a closely spaced parallel configuration, a “V” configuration in which the spacing between DNA molecules is small on one end and variable but larger on the other, and a “U” geometry in which the DNA molecules on both ends are spaced at lager separations than in the close spaced geometry. The authors measure the torque associated with braiding the DNA to extract the twist persistence length, Ceff, the critical buckling torque at which the braided DNA forms a four strand plectoneme, and the change in extension per turn in the plectonemic regime. For the situations with larger separations on one or both sides the authors observe a torque overshoot associated with the initial turns (less than +/- 0.5 turns) and a discontinuity in the torque as the number of turns are increased beyond 0.5. Based on these measurements the authors speculate on the partitioning of supercoiling on ahead of and behind replicating DNA for different separations of the DNA exiting the replisome.

The measurements are well done and the development of the DNA constructs to achieve the different braiding geometries are a useful contribution to the field. However, the main results and broader implication of the results concerning the partitioning of supercoiling with respect to the replisome have largely if not completely been anticipated by previous work form the author’s lab (figure 5 of reference 4) and from other labs (primarily but not exclusively reference 23). Together these previous publications demonstrate the differences in torsional compliance between braided and duplex DNA, completely anticipating the central results of this work, and elaborate the relevant calculations for the torque overshoot, the torque jump and more generally the mechanics of DNA braiding. The results presented in this work therefor do not represent an important advance in our understanding of the mechanics of the braiding of DNA molecules. I think that this work is solid and, baring some minor points raised below, is correct with the conclusions well supported by the data, however the overall contribution to the field warrants publication in an excellent but more specialized journal – for example the Biophysical Journal where reference 23 appeared. This work is of comparable import and interest.

Minor points:
1. All of the data presented represent the averages over many measurements. It would be useful to present representative individual measurements of the torque and extension for the different geometries. It would also be helpful to include a measure of the statistical uncertainties in the averaged data.
2. For the “V” and “U” geometries, it seems that the geometric fits to the extension as a function of rotation (equations 1, 2, and 3 in the SI) are sufficient to obtain estimates of the “a” and “b” separation parameters, which are not provided in the text. Estimates of the “a” and “b” parameters are important in understanding the range of parameters obtained and in interpreting the results. These parameters are crucial for understanding the averaged torque and rotation data for the U and V geometries in figure 3 a and b. There is no discussion of the criteria used to group tethers with different geometries for averaging. What are the ranges of a and b parameters and the uncertainties of these parameters that are included in the averages. In a related point, it seems that it would be better to present the measurements for each tether geometry separately rather than averaging the data together. Can the authors provide the data for individual tethers?
3. Can the authors provide the dimensions of the quartz cylinders? In particular the diameter of the cylinder would be helpful to understand the range of possible “U” geometries.
4. Can the authors discuss the possibility of asymmetric tethers. Although the cartoons show symmetric tether geometry with respect to the axis of the cylinder the general case cold result in tethering of the DNA, particularly in the “O” and “V” geometries anywhere across the face of the cylinder. Naively one might expect that the location of the tether point relative to the rotation axis of the cylinder could introduce an additional torque component or an error in the measured torque signal. Can the authors address this potential concern with the random attachment of the DNA to the cylinder face and the effects of this attachment being offset from the rotation axis of the cylinder?
5. Can the authors provide the number of molecules averaged to obtain the data in Figure 3 c and d and as discussed above give a sense of the range of a and b parameters included in the averages for each point? In a related point, it would be helpful to include examples of the V and U substrate braiding experiments at 2 pN, the force at which all of the measurements in figure 3 c and d were performed. The examples in Figure 3 a and b were obtained at significantly higher forces.
I hope that these questions and comments will aid the authors in revising this work for publication. I think that overall the work is well-done and the data are of high quality, but it represents a modest advance over prior work published by the authors and others.

(Remarks on code availability)

Version 1:

Reviewer comments:

Reviewer #1

(Remarks to the Author)

The authors have done a commendable job addressing the reviewers' comments and incorporating substantial revisions into the manuscript. The addition of the refined Monte Carlo simulation framework and the new single-DNA tether experiments strengthen the study, while revisions throughout the text better position the findings within the context of prior work.

Despite these improvements, the study remains primarily a refinement and extension of existing understanding rather than a fundamentally new conceptual advance. As noted by several reviewers, the reported results are largely consistent with expectations arising from previous single-molecule investigations and theoretical models of DNA braiding. Furthermore, the proposed biological significance of the observed torque overshoot remains speculative and is not directly demonstrated.

In my view, this is a rigorous and technically strong study that would be well suited for a specialized journal where the experimental methodology, computational modeling, and quantitative analysis can be more fully appreciated. I agree with the suggestion of journals such as Biophysical Journal or, if the authors wish to remain within the Nature Portfolio, Scientific Reports.

(Remarks on code availability)

Reviewer #2

(Remarks to the Author)
As I argued in more detail in my previous review, these are novel and important measurements that will be valuable to the field. The study is further strengthened by the inclusion of additional supporting data and by the new MC simulations. There are aspects of the analysis and conclusions that remain unjustified and should be amended or removed — but the work is otherwise appropriate for publication in Nature Communications.

To clarify a point: the observed torque overshoot is a property of the equilibrium torque(n) relationship measured in the fixed-n ensemble. In the revised manuscript, this result is further clarified by the inclusion of forward-and-reverse torque(n) curves (Fig. S4) and by MC simulations to probe the equilibrium relationship in this ensemble. If the braid is placed in series with a soft torsional spring such as single DNA duplex (as in Fig. S5), then fluctuations in braid catenation will become accessible, with behavior approaching the fixed-torque ensemble for a sufficiently soft spring — please see my previous comments for additional details. In Fig. S5 and the associated sections of the text, the authors assume without justification that braid catenation in the forked molecule is completely suppressed for torques below the overshoot torque (as defined at fixed-n for the isolated braid). This is now described as a “kinetic barrier” but there is no description or elaboration of a kinetic model or discussion of timescales, and Fig, S5 implies (without explanation) that rates of crossing the barrier are negligible at all relevant timescales and at all torques considered. As the authors note, the geometry in Fig. S5 is in any case fairly contrived for biological contexts — but if they are going to devote space to predicting the behavior of this geometry, it should be better justified or reconsidered. In discussing the overshoot, the authors may also miss an opportunity to relate the phenomenon to previous measurements and models of torque overshoots in DNA structural transitions, including past work from the authors' lab on plectonemic buckling.

Additional comments: more details should be provided for the MC simulations, including how parameters were chosen, how results were scaled for comparison with experiments, and how torque curves appear for low catenation number in v-substrates (how closely is the experimental “torque gap” feature recapitulated?). I would also recommend displaying representative chain configurations.

(Remarks on code availability)

Reviewer #3

(Remarks to the Author)
I appreciate the significant additional effort the authors put into revising and extending the MC simulations and the additional insights obtained by combining the MC simulation results with the experimental data. The new insights combined with the new tools made available to the community have significantly increased the importance and impact of this work.
The authors have addressed my questions and concerns in their revived manuscript. All together I recommend acceptance.

(Remarks on code availability)
The code includes a reasonable readme and appears to be complete. It could use some additional comments, but overall the code is sound and it is a useful translation of the original code into matlab, which will make it more user friendly and widely deployable.

Open Access This Peer Review File is licensed under a Creative Commons Attribution 4.0 International License, which permits use, sharing, adaptation, distribution and reproduction in any medium or format, as long as you give appropriate credit to the original author(s) and the source, provide a link to the Creative Commons license, and indicate if changes were made.
In cases where reviewers are anonymous, credit should be given to 'Anonymous Referee' and the source.
The images or other third party material in this Peer Review File are included in the article's Creative Commons license, unless indicated otherwise in a credit line to the material. If material is not included in the article's Creative Commons license and your intended use is not permitted by statutory regulation or exceeds the permitted use, you will need to obtain permission directly from the copyright holder.
To view a copy of this license, visit https://creativecommons.org/licenses/by/4.0/

Response to Reviewers

Re: Manuscript number: Decision on manuscript NCOMMS-25-06808-T
Geometry of Braided DNA Dictates Supercoiling Partitioning by Hong et al.

We greatly appreciate the time and effort of all three Reviewers and sincerely thank them for their thoughtful and constructive comments. Below, we will begin with a summary of major changes to figures, followed by a point-by-point response, with each comment (bold) followed by our response (not bold). In the manuscript files, we have also highlighted the important changes in red.

Summary of Major Changes to Figures

1. Reviewer #2 and Reviewer #3 suggested that we more rigorously examine the assumptions of the geometric model and use the model to guide the interpretation of the braiding geometry and braiding torsional mechanics. Because the geometric model relies on significant assumptions, it does not accurately predict measurements once braiding initiates and cannot predict braid buckling. To circumvent this significant drawback, we have been working extensively to develop a Monte Carlo (MC) simulation approach to accurately predict DNA braiding. In the revised manuscript:
    a. Figures 2 and 3 have been expanded to show the MC simulation results, which are in excellent agreement with measurements.
    b. Figure 4 is new and shows how the MC simulations can be used to guide the interpretation of the braiding geometry and braiding torsional mechanics.
    c. Figure S7 is new and shows how the predictions of the geometric model deviate significantly from measurements.

2. Reviewer #2 suggested that we measure the torsional mechanical properties of a single DNA substrate under different forces using the same conditions as those for the braids. We have conducted these new experiments. The results are now shown in Figure 2b,c,d of the revised manuscript.

3. Reviewer #1 and Reviewer #2 commented on the relevant end-separation distances *in vivo*. We have highlighted that the 'O substrate' is most relevant to the *in vivo* replication *c*onfiguration. For the two remaining substrates, we have now clarified that the 'V substrate' is more relevant than the 'U substrate'. We have revised Figure 5 to show configurations of both the 'O substrate' and 'V substrate'.

4. Reviewer #2 asks us to show torque-twist curves taken in both directions (unwinding/rewinding) in order to clearly establish whether the overshoot features are representative of the equilibrium torque-twist relationship. We now show these measurements in a new SI figure, Figure S4, of the revised manuscript.

Reviewer #3 asks us to show individual traces of torsional measurements for each substrate. We now show three individual traces for each of the three substrates in a new SI figure, Figure S1, of the revised manuscript. For each trace, we have also indicated the end separation distances a and b by comparing measurements and simulations.

Reviewer #1 (Remarks to the Author):

In the report by Hong et al. entitled 'Geometry of Braided DNA Dictates Supercoiling Partitioning' the authors describe a sophisticated optical trapping technique that permits direct measurement of torque during the braiding of two double-stranded DNA molecules. DNA braids are employed to model the behavior of DNA catenanes that form in vivo as replication intermediates. Extensive literature from the past half century has characterized the topological properties of DNA during replication and the propensity of DNA to form braid-like structures as a consequence of torsional strain that accumulates ahead of a progressing replication fork. The physics of DNA braiding and the impact of DNA braid chirality on the catalytic activities of replication-associated enzymes has been reported over the past twenty years.

The main advance of the present work is the well-controlled braiding geometry that the authors achieve using a clever molecular biology approach to modify the DNA molecules used in the study. The approach permits them to generate O-braides, where the DNA ends are relatively close, on the order of one persistence length of dsDNA (~150nm), V-braids where one set of ends are close and the others are much further apart, and U-braids where both sets of ends are far apart. The authors relate the O-braid separation to the spacing that might be expected for DNAs associated with the actively progression replication machinery - however, this distance is likely to be much smaller than the end separation in the O-braid geometry.

We appreciate the comment on the end separation distance relevant to an active replication machinery. This is indeed a question we have been curious about for some time. Based on the structures of the eukaryotic and prokaryotic replisomes[1–5], we estimate the replisome leads to an end separation 20-50 nm. In our O-template design, the end of the substrate contains a multi-tagged region of 69 or 70 bp, close to that at the replisome. As the Reviewer noted, DNA may resist bending, which increases the effective end separation. We estimate that each multi-tagged anchoring region yields an effective DNA end separation of approximately 42 nm (see Methods). This value accounts for the contributions of DNA bending on both sides of the anchoring region and is comparable to that estimated at the replisome. We have now detailed the method to estimate this distance on pages S8-S9 of the revised manuscript.

We have also clarified the dimensions of a replisome and our designs on pages 2 and 4 of the revised manuscript. We have now further clarified that the 'O substrate', which has small end separations at both ends, should best resemble the *in vivo* geometry of daughter strands during replication on page 5 of the revised manuscript.

The authors report a detailed biophysical characterization of the braiding behavior of each type of braid, and in particular the specific optical trapping technique developed years ago in the author's lab allows direct measurement of the braiding density - torque relationship. Previous single molecule studies of DNA braids by multiple research groups arrived at somewhat similar conclusions to the present work, but relied on simulations and geometric models to extract information about the torque response of DNA braids.

There is no doubt that the present work provides a higher resolution view and detailed torque response characterization of DNA braids with different geometries - showing that when two of the DNA ends are far apart there is a significant torque overshoot that occurs during the initial formation

of the first DNA braid crossing. In other words, the energetic penalty for collapsing two DNA molecules with ends that are anchored far from each other is much larger than when the ends are close. This single molecule response was reported previously for less controlled DNA braiding systems, but the specific torque characterization in this study is an advance from the prior work.

We thank the reviewer for recognizing that the more controlled braiding geometries and direct torque measurements in our study represent an advance from prior studies.

While the authors cite all of the appropriate literature in this current manuscript, they do not place the current work sufficiently into the context of the previous single molecule studies so as to highlight how their results represent a significant advancement. Moreover, while the authors speculate that braid geometry may represent a novel mode of regulation of DNA structures in vivo, they do not perform any experiments that directly support or test this interesting hypothesis. Thus, the work by Hong et al. is a more biophysically focussed study whose biological relevance remains to be appropriately explored. For this reason, it is not clear that the well-described study of torque-dependence of DNA braiding on different geometries will be of sufficient interest to a broad readership to justify its publication in Nature Communications.

Upon reading this comment from the Reviewer, we recognized that our original manuscript did not sufficiently clarify the novelty of this work. We thank the Reviewer for this comment. In this response, we have substantially revised the introduction to better position our study within the context of previous single-molecule studies on pages 2-3 of the revised manuscript. We have now clarified that elucidating how daughter-strand geometry affects DNA supercoiling partitioning requires direct torque measurements on braiding DNA substrates with defined end separations (20-50 nm). This feat, which we have achieved in the current study, was not possible in prior single-molecule studies. In addition, to our knowledge, there have been no explicit discussions recognizing the significance of braiding geometry in the partitioning of DNA supercoiling during replication. Therefore, there are significant knowledge gaps in these studies.

As the Reviewer noted, prior studies relied on simulations and geometric models to extract information about the torque response of DNA braids. However, the geometric model does not accurately describe torsional properties after braiding begins and does not predict braid buckling. Thus, the geometric model cannot provide guidance on how braiding geometry affects the torque gap, the effective torsional modulus of a braid, and the braid buckling. To illustrate this, we have added a new SI figure, Figure S7, to the revised manuscript to compare our measured extension-versus-turns and torque-versus-turns relations with those predicted by the geometric model. Figure S7 clearly shows a substantial deviation between predictions and measurements once braiding starts. These limitations greatly restricted our ability to interpret the data using the geometric model.

Thus, we decided to perform Monte Carlo (MC) simulations to investigate the torsional response of braided DNA. Although Monte Carlo simulations have previously been used to study DNA braiding[7], predictions of these simulations deviate substantially from our measurements as the catenation number increases (Figure R1 below in this response letter). Thus, it was necessary to refine the approach to improve accuracy and achieve quantitative agreement between simulation results and measurements. We therefore extended the model to incorporate a stronger topological invariant to constrain topology and a Debye-Hückel electrostatic interaction potential instead of a hard-wall repulsion between DNA strands (a parameter that plays a key role in determining the braid diameter), as described on pages S5-

S9 of Methods in the revised Supplementary Information. These extensions are essential at higher catenation numbers, especially after buckling occurs.

The addition of our MC simulation results prompted a major restructuring of the manuscript to directly compare experimental measurements with theoretical predictions and to use the simulations to guide data interpretation. As demonstrated in the revised Figure 2a,c,d, revised Figure 2a,b, and new Figure 4, this theoretical framework has greatly improved our understanding of the role of braiding geometry in torsional mechanics. These additions have been highlighted throughout the revised manuscript. We believe these changes have significantly enhanced both the content and the novelty of the manuscript.

As the Reviewer noted, it would be interesting to investigate whether braiding geometry represents a novel mode of regulating DNA structures and replication *in vivo*. Although testing this hypothesis is beyond the scope of the current study, we hope to pursue such *in vivo* studies in the future.

Reviewer #2 (Remarks to the Author):

In this manuscript, Hong et al. measure the torsional response of braided DNA. The measurements in this study provide a significant advance beyond prior work on this topic, by using more defined DNA substrates than were previously employed and by collecting detailed datasets with direct torque measurements. The measurements are relevant to understanding the behavior of intertwined DNA duplexes in biology, including during replication where supercoiling can partition in front and behind the fork (as the authors emphasize and as Wang and coworkers previously investigated in elegant working using chromatinized DNA). The measurements are also of general interest in understanding the physics of intertwined polymers, and provide valuable data for comparison with theoretical treatments. The questions pursued in this study are thus important, and the measurements are a novel and substantial contribution. However, the authors should re-examine their analysis and conclusions before the study can be published.

We thank this Reviewer for recognizing the importance of our prior and current work on DNA braiding and for the thoughtful and constructive feedback. We fully agree with the need to re-examine our analysis and conclusions and have worked extensively to address this concern (see below).

In Figure 5b, supported by Table S2, the authors model a forked DNA molecule under conditions where torque(n) measurements of a braid produce a torque "overshoot". They assume that no supercoiling can partition into the braid at torques below the overshoot. This will not in general be true for an equilibrium treatment of a braid in series with a single torsionally constrained DNA molecule. The torque overshoot is measured in an experiment where torque(n) is obtained in the fixed-n ensemble, and the result is a nonmonotonic function where the same torque is reached at multiple values of n. To model the forked system, a statistical mechanical treatment is needed that considers all possible values of the fluctuating braid catenation number (n) while fixing the total supercoiling across the forked DNA including the single-DNA section. In the limit of a long (and hence torsionally soft) appended single-DNA section, the behavior of the braid section will approach the fixed-torque ensemble — where the distribution P(n) at a given fixed torque will be bimodal for a range of torques below the overshoot torque, as implied by the shape of the nonmonotonic torque(n) measured above. See previous work on torque "overshoots" in plectonemic DNA, B-Z transitions, etc. The above assumes an equilibrium treatment, which I think was the implied intent of the authors; if

they wish to propose a *kinetic* barrier to braid initiation then this should be explicitly described, motivated, and justified.

We very much appreciate this comment on the barrier nature of the “torque overshoot”. We are grateful to the Reviewer for providing a detailed and thorough explanation of the comment. As the Reviewer noted, the proper thermodynamic ensemble relevant to replication is the fixed-torque ensemble, since the torque is the same for the parental DNA and the daughter DNA strands. Therefore, the braid's torque overshoot can only prevent the start of braiding kinetically and does not represent the equilibrium torque. Therefore, in the context of replication, this torque barrier is indeed a “kinetic barrier”. We really like the use of this terminology, which concisely captures the nature of the barrier. We have now adopted this term and explicitly state it on pages 8 and 10 in our revised manuscript.

Related to the above, when investigating partitioning across the fork, the authors should discuss the relationships of the regimes they consider to plausible biological conditions during replication. At a replication fork, it is not immediately clear how the DNA separation at *both* ends of the braid can be large (as modeled in 5b), given the relatively small size of the replication machinery. For all modeled conditions (Fig 5ab, Fig S5), the authors consider a regime of moderate tension (2 pN) and moderate supercoiling where buckling does not occur. In the cell, lower tensions where buckling occurs are likely to be an important regime. (In fact the DNA is drawn as buckled in the fork cartoon provided by the authors in 5b, a picture that is not representative of the conditions modeled in partitioning calculations.) The partitioning between the single-DNA and the braid can be quite different in a regime where the DNA buckles. The authors may consider expanding the regimes investigated.

The Reviewer inquired about biological conditions under which the DNA separation at both ends of the braid can be large. In the original manuscript, we envisioned that this could occur at one end when SMC proteins fail to bring the two daughter strands together. At the replisome end, this could occur when the replisome encounters significant replication stress (such as a DNA lesion or a collision with a head-on RNA polymerase), leading to the recruitment of a host of DNA repair proteins for fork stabilization, repair, and remodeling, and thereby enlarging the daughter DNA separation. Because this configuration occurs only under specialized conditions, we have decided to place less emphasis on this scenario and have moved the discussion of its implications for DNA supercoiling partitioning to Figure S5 of the revised manuscript. Instead, in the revised Figure 5b, we now discuss the scenario when only one end of the braid is large. This could occur when the daughter strands fail to be brought together at the other end by SMC proteins but are brought together at one end by the replisome, so that the braiding geometry resembles the ‘V substrate’.

As the Reviewer noted, DNA may buckle under increased torsional stress, with the buckling transition depending on DNA tension. Thus, DNA buckling will inevitably change how DNA supercoiling partitions during replication. *In vivo*, DNA may be under tension due to forces generated by motor proteins; for example, RNA polymerases have been found to generate forces on the order of tens of pN[8,9]. For the ‘O substrate’ and the single-DNA substrate, we have investigated how this buckling transition depends on the DNA tension between 0.5 and 3 pN. As shown in Figure 2c,d of the revised manuscript, the effective twist persistence length shows only slight dependence on force, while the buckling more strongly depends on force. This allows prediction of how supercoiling partitioning changes with force, under the 2 pN condition shown in Figure 5a of the revised manuscript. At low superhelical density, supercoiling partitions primarily behind the replisome, with partitioning not strongly dependent on force. As the superhelical density increases, the single-DNA substrate at the front buckles, and this buckling occurs at

higher superhelical densities with increasing force. We have not measured the torsional mechanics of the ‘V substrate’ and ‘U substrate’ at forces other than 2 pN.

In the revised manuscript, we have followed this Reviewer's suggestion and expanded the discussion of DNA supercoiling partitioning to account for the possibility of DNA buckling, which occurs only at high DNA superhelical density when topoisomerase activities are low. These are shown in Figure 5a (relevant to the ‘O substrate’), Figure 5b (relevant to the ‘V substrate’), and Figure S5 (relevant to the ‘U substrate’).

Experimentally, the authors should show torque-twist curves taken in both directions (unwinding/rewinding) in order to clearly establish whether the overshoot features are representative of the equilibrium torque-twist relationship.

We have taken this suggestion from the Reviewer and have shown the torque-versus-turns curve of the overshoot in both directions (adding or removing turns) as a new SI figure, Figure S4 of the revised manuscript. As shown in this figure, the two curves nearly coincide, indicating that the measurements were performed under a condition close to equilibrium.

Another experimental point that should be clarified is the topology of the V substrate. The construction method implies that one of the arms will be torsionally constrained, and the construct is described that way in the Methods. In the main text it is stated that both arms are nicked before the measurement, but it should be explained how this is ensured — or whether and how tethers are selected for serendipitous cases where both arms are unconstrained.

The ‘V substrate’ was prepared with both strands nicked to mimic the daughter strand topology during replication. We previously surveyed the literature about whether each of the two strands can rotate freely around its own helical axis during replication[10]. There is a general agreement that the lagging strand can rotate freely around its helical axis due to the presence of regions of the Okazaki fragments. There is evidence that the lagging strand can also rotate freely around its helical axis: in a divergent replication, the leading strand is at the lagging strand of the other replisome; DNA polymerases may transiently detach from the DNA and/or rotate relative to the helicase at the replication fork. Thus, we have nicked both strands of the ‘V substrate’ so that each daughter strand can rotate freely around its own helical axis.

Previously, we systematically studied the efficiency of nicking enzymes and found that 30 U/mL Nt. BsmAI (NEB, R0121) for 10 min nicked 100% of a template containing 9 nicking sites (see Extended Data Figure 5c of reference[11]). In this work, we also used 30 U/mL Nt. BsmAI (NEB, R0121) for 10 min to nick a DNA template containing 12 nicking sites. Under this condition, this template should be essentially fully nicked. We have clarified this on page S4 of the Methods section in the revised Supplementary Information.

In general the authors should be careful to clearly and accurately describe the steps and assumptions in the derivation of theoretical expressions, even when they closely follow previous work. E.g. the supplement implies that eq. 5 follows directly from eq. 2 via a Maxwell relation, which is not the case. A longer discussion of the limitations of the geometrical model would be appropriate, including plotting eq. 1-2 and 4-5 overlaid on plots of extension and torque vs supercoiling.

We appreciate these suggestions. We have significantly expanded the discussion of the derivation of the geometric model on pages S9-S11 of the Methods section in the revised Supplementary Information. This expansion allows us to state more accurately the assumptions required to obtain analytical expressions.

As we discussed on pages 8-10 of the revised manuscript, while the geometric model accurately predicts torsional properties before braiding initiates (within ±0.5 turns), it does not accurately describe torsional properties once braiding begins and cannot predict the buckling behavior. Thus, the geometric model cannot provide guidance on how braiding geometry affects the torque gap, the effective torsional modulus of a braid, and the braid buckling. To more clearly illustrate this, as suggested by the Reviewer, we have added a new SI figure, Figure S7, to the revised manuscript to compare our measured extension-versus-turns and torque-versus-turns relations with those predicted by the geometric model. Figure S7 shows a substantial deviation between predictions and measurements once braiding begins, due to the assumptions used to derive an analytical expression for the geometric model.

These assumptions are substantial, raising concerns about the model's validity. This motivated us to develop a Monte Carlo simulation approach to investigate how braiding geometry affects the torsional properties of braids. Although Monte Carlo simulations have previously been used to study DNA braiding[7], the predictions of these prior simulations deviate from our measurements (Figure R1 below in this response letter). Thus, it was necessary to refine the approach to improve accuracy and achieve quantitative agreement between simulation results and measurements. We therefore extended the model to incorporate a stronger topological invariant to constrain topology and a Debye-Hückel electrostatic interaction potential instead of a hard-wall repulsion between DNA strands (a parameter that plays a key role in determining the braid diameter), as described on pages S5-S9 of Methods in the revised Supplementary Information. These extensions are essential at higher catenation numbers, especially after buckling occurs.

We found excellent agreement between simulations and measurements, as shown in Figures 2a and 3a,b of the revised manuscript. Therefore, the simulations can now be used to provide guidance for understanding how the geometry (end separation distances a and b) of braiding substrates affects torsional parameters, such as torque overshoot, torque gap, torsional modulus, and buckling torque. These new results are shown in Figure 4 of the revised manuscript.

Limitations of the experiment should also be discussed at more length — while substrates are more well-defined than previous work, the end separation is still not controlled for the open ends of V and U substrates; single-DNA results for comparison with braids rely on past literature rather than side-by-side comparisons under the same conditions; forked DNA is modeled rather than directly experimentally measured; and limited torque resolution seems to preclude detecting smaller overshoots or clearly characterizing the shape of the torque response during the first half turn.

We thank the Reviewer for succinctly summarizing the discussion points for the limitations of our work. The first two limitations should have been reasonably addressed.

The Reviewer correctly noted that the 'V substrate' and 'U substrate' do not have fully controlled geometry. We have addressed this limitation using Monte Carlo simulations. The end separation distances a and b give rise to detectable features in the extension-versus-turns and torque-versus-turns relations, which can be extracted by comparing the measured relations with those from simulations (see Figure 2a and Figure 3a,b of the revised manuscript). We validated that this treatment works well by

comparing the torsional parameters from measurements with those from simulations (see Figure 4c,d of the revised manuscript). This treatment, albeit a bit indirect, allows us to estimate the end separation distances a and b of a broad range of geometries. In addition, we have added a new SI figure (Figure S1) to the revised manuscript to show individual traces of the three substrates. For each trace, we have also indicated the values of a and b by comparing measurements and simulations.

The Reviewer also noted that our initial manuscript included single DNA-substrate results from the previous literature rather than measurements under the same conditions. We have addressed this limitation by measuring the torsional mechanical properties of a single DNA substrate under different forces using the same conditions as those for the braids. These measured extension-versus-turn and torque-versus-turn relations are now shown in Figure 2b of the revised manuscript. For completeness, we also show the corresponding predictions using the Monte Carlo simulations. We find a good agreement between measurements and simulations. The features of these relations, such as the effective torsional modulus, the buckling torque, and the superhelical density at buckling, all as a function of force, are also shown in Figures 2c,d of the revised manuscript. This allows for a direct comparison of the single and double substrates.

The remaining two limitations, namely, that forked DNA is modeled rather than directly experimentally measured, and the limited torque resolution precludes detecting smaller overshoots or clearly characterizing the shape of the torque response during the first half turn, are now discussed under Discussion on pages 11-12 of the revised manuscript.

Minor: on page 9, "the torque overshoot and the torque gap are proportional…" — I think the authors mean "hat tip" and not "torque gap".

We thank the Reviewer for catching this typo. This sentence was removed because we now rely on the Monte Carlo simulations for theoretical guidance in the revised manuscript.

The authors may miss an opportunity for detailed comparisons with more sophisticated theory; it is reasonable for this to be left for future work, but will be important for the authors to provide accessible data for others to make use of these important new experimental results.

We fully agree that more detailed comparisons with a sophisticated theory would be valuable, which motivated us to develop the Monte Carlo simulation approach. These simulations have now provided important theoretical insight into how geometry influences braiding torsional mechanics. We are grateful that the Reviewer's comment has inspired us to pursue this effort.

Reviewer #3 (Remarks to the Author):

Hong and co-authors employ the angular optical trap (AOT) to measure the torque associated with "braiding" or wrapping to double stranded DNA molecules around each other, akin to precatenane formation occulting in daughter DNA molecules following replication. The authors develop different DNA constructs to tether the optically trapped quartz cylinder to the surface in three different geometries: a closely spaced parallel configuration, a "V" configuration in which the spacing between DNA molecules is small on one end and variable but larger on the other, and a "U" geometry in which the DNA molecules on both ends are spaced at lager separations than in the close spaced geometry.

The authors measure the torque associated with braiding the DNA to extract the twist persistence length, Ceff, the critical buckling torque at which the braided DNA forms a four strand plectoneme, and the change in extension per turn in the plectonemic regime. For the situations with larger separations on one or both sides the authors observe a torque overshoot associated with the initial turns (less than +/- 0.5 turns) and a discontinuity in the torque as the number of turns are increased beyond 0.5. Based on these measurements the authors speculate on the partitioning of supercoiling on ahead of and behind replicating DNA for different separations of the DNA exiting the replisome.

The measurements are well done and the development of the DNA constructs to achieve the different braiding geometries are a useful contribution to the field. However, the main results and broader implication of the results concerning the partitioning of supercoiling with respect to the replisome have largely if not completely been anticipated by previous work form the author's lab (figure 5 of reference 4) and from other labs (primarily but not exclusively reference 23). Together these previous publications demonstrate the differences in torsional compliance between braided and duplex DNA, completely anticipating the central results of this work, and elaborate the relevant calculations for the torque overshoot, the torque jump and more generally the mechanics of DNA braiding. The results presented in this work therefor do not represent an important advance in our understanding of the mechanics of the braiding of DNA molecules. I think that this work is solid and, baring some minor points raised below, is correct with the conclusions well supported by the data, however the overall contribution to the field warrants publication in an excellent but more specialized journal – for example the Biophysical Journal where reference 23 appeared. This work is of comparable import and interest.

We thank the reviewer for noting the quality of our measurements, the rigor of our conclusions, and the usefulness of our DNA constructs. We agree that reference 23 of the original manuscript, along with other studies, has made important contributions to understanding DNA braiding mechanics. However, we do not think that our main results and broader implications have been "completely anticipated" by previous work. As discussed in this revised manuscript, understanding how braiding geometry affects the torsional properties of braids requires 1) braiding substrates of defined geometry and 2) theoretical guidance for the interpretation of measurements.

Our work presents the first direct measurements of DNA braiding torques under a well-defined geometry. We achieved this using a novel 'O-substrate'. Our 'V-substrate' has one end with a well-defined end separation, providing another valuable configuration for exploring how geometry influences braiding torsional mechanics. Our results suggest that the presence of a torque gap can significantly affect the initiation of fork rotation during replication.

Accurate theoretical guidance on how braiding geometry affects the torsional properties of braids has been lacking. In our initial manuscript, we relied on the geometric model, which works well before braid formation (within ±0.5 turns), but does not accurately describe torsional properties after braiding begins and does not predict braid buckling. Thus, the geometric model cannot provide guidance on how braiding geometry affects the torque gap, the effective torsional modulus of a braid, and the braid buckling. To illustrate this, we have added a new SI figure, Figure S7, to the revised manuscript to compare our measured extension-versus-turns and torque-versus-turns relations with those predicted by the geometric model. Figure S7 clearly shows a substantial deviation between predictions and measurements once braiding starts. These limitations greatly restricted our ability to interpret the data using this model.

During manuscript revision, concerns about the geometric model's limitations motivated us to develop an enhanced Monte Carlo simulation approach to investigate how braiding geometry affects the torsional properties of braids. Although Monte Carlo simulations have previously been used to study DNA braiding[7], the predictions of these simulations deviate from our measurements (Figure R1 below in this response letter). Thus, it was necessary to refine the approach to improve accuracy and achieve quantitative agreement between simulation results and measurements. We therefore extended the model to incorporate a stronger topological invariant to constrain topology and a Debye-Hückel electrostatic interaction potential instead of a hard-wall repulsion between DNA strands (a parameter that plays a key role in determining the braid diameter), as described on pages S5-S9 of Methods in the revised Supplementary Information. These extensions are essential at higher catenation numbers, especially after buckling occurs. We found excellent agreement between simulations and measurements, as shown in Figures 2a and 3a,b of the revised manuscript. Therefore, the simulations provide guidance for understanding how the geometry (end separation distances *a* and *b*) of braiding substrates affects torsional parameters, such as torque overshoot, torque gap, torsional modulus, and buckling torque. These new results are shown in Figure 4 of the revised manuscript.

We appreciate the chance to clarify the novelty of our work. We hope that our revised manuscript clearly conveys the advances of this study.

Minor points:

1. All of the data presented represent the averages over many measurements. It would be useful to present representative individual measurements of the torque and extension for the different geometries. It would also be helpful to include a measure of the statistical uncertainties in the averaged data.

We have taken this suggestion from the Reviewer and added a new SI figure, Figure S1, to show nine individual traces of extension and torque as a function of turns added at 2 pN, with three traces for each of the 'O-substrate', the 'V-substrate', and the 'U-substrate'. These individual traces should give an indication of measurement uncertainties.

To further get the sense of measurement uncertainties, we can examine the measurements from the 'O substrate', which show a hat slope of 1.42 ± 0.16 nm/turns (mean ± SD) and a torque gap of 2.28 ± 0.70 pN·nm (mean ± SD) as indicated in the first data point in the right plot of Figure 4c of the revised manuscript. The 'V-substrate' and the 'U-substrate' have larger uncertainties because traces with somewhat different geometries were grouped together.

2. For the "V" and "U" geometries, it seems that the geometric fits to the extension as a function of rotation (equations 1, 2, and 3 in the SI) are sufficient to obtain estimates of the "a" and "b" separation parameters, which are not provided in the text. Estimates of the "a" and "b" parameters are important in understanding the range of parameters obtained and in interpreting the results. These parameters are crucial for understanding the averaged torque and rotation data for the U and V geometries in figure 3 a and b. There is no discussion of the criteria used to group tethers with different geometries for averaging. What are the ranges of a and b parameters and the uncertainties of these parameters that are included in the averages. In a related point, it seems that it would be better to present the measurements for each tether geometry separately rather than averaging the data together. Can the authors provide the data for individual tethers?

Just as the Reviewer noted, it should be possible to estimate the values of a and b for each trace. We fully agree with the Reviewer that the extension-versus-turns and torque-versus-turns data should contain information for estimating the end-separation distances a and b using an appropriate model. As shown in the new SI figure (Figure S7) the geometric model predicts extension and torque that deviate substantially from the measurements, indicating that this model is not suited for reliably estimating a and b. In contrast, our MC simulations reproduced extension and torque in agreement with the measured values for all three substrates, as demonstrated in the revised Figures 2a and 3a,b.

Therefore, we can now use our MC simulations to estimate a and b of each trace in the revised manuscript. To illustrate this operation, for each measured trace shown in Figure S1 of the revised manuscript, we have now provided estimates of a and b guided by our MC simulations. This is done for nine example traces with three for each of the 'O substrate', 'V substrate, and the 'U substrate'.

Furthermore, we have devoted a new figure (Figure 4) to the revised manuscript to address this concern. In this figure, we have used the MC simulations to estimate a and b, reporting them in terms of $a \cdot b$ and $a + b$ for measurements made on the three substrates at 2 pN. Traces with similar torque gap or torque overshoots were grouped for averaging to reduce uncertainties, and the error bars on the estimated $a \cdot b$ and $a + b$ should provide a quantitative measure of their uncertainties.

3. Can the authors provide the dimensions of the quartz cylinders? In particular the diameter of the cylinder would be helpful to understand the range of possible "U" geometries.

We thank the Reviewer for spotting this accidental omission. The dimensions of the quartz cylinders are now indicated on page S3 of the Supplementary Information of the revised manuscript.

4. Can the authors discuss the possibility of asymmetric tethers. Although the cartoons show symmetric tether geometry with respect to the axis of the cylinder the general case cold result in tethering of the DNA, particularly in the "O" and "V" geometries anywhere across the face of the cylinder. Naively one might expect that the location of the tether point relative to the rotation axis of the cylinder could introduce an additional torque component or an error in the measured torque signal. Can the authors address this potential concern with the random attachment of the DNA to the cylinder face and the effects of this attachment being offset from the rotation axis of the cylinder?

The Reviewer is correct that a braiding substrate may attach to the bottom of the cylinder off-center, causing some wobbling as the cylinder is rotated, leading to angle-dependent signals in the measurements. During measurements, we recorded only tethers that showed little angle-dependent signal, consistent with good centering. Even so, we could not rule out the possibility of some slight off-centering, which could introduce some uncertainties in the measurements.

5. Can the authors provide the number of molecules averaged to obtain the data in Figure 3 c and d and as discussed above give a sense of the range of a and b parameters included in the averages for each point? In a related point, it would be helpful to include examples of the V and U substrate braiding experiments at 2 pN, the force at which all of the measurements in figure 3 c and d were performed. The examples in Figure 3 a and b were obtained at significantly higher forces.

The data presented in Figure 3c,d of the original manuscript are now integrated into Figure 4c,d of the revised manuscript. We have now indicated the number of molecules in the figure legends.

The traces shown in Figure 3a,b of the original manuscript were indeed performed at 2 pN (instead of higher forces). These traces are again shown in Figure 3a,b of the revised manuscript. Please see the figure legend where the force value is indicated.

I hope that these questions and comments will aid the authors in revising this work for publication. I think that overall the work is well-done and the data are of high quality, but it represents a modest advance over prior work published by the authors and others.

We greatly appreciate the reviewer's thoughtful comments, which we found very helpful, and we are grateful to this Reviewer for their careful and constructive review, which has improved the manuscript. In response to these suggestions, we have explicitly highlighted the novelty of the work relative to prior studies by adding a dedicated paragraph in the Introduction and clarifying the unique methodological and analytical approaches in the Results section.

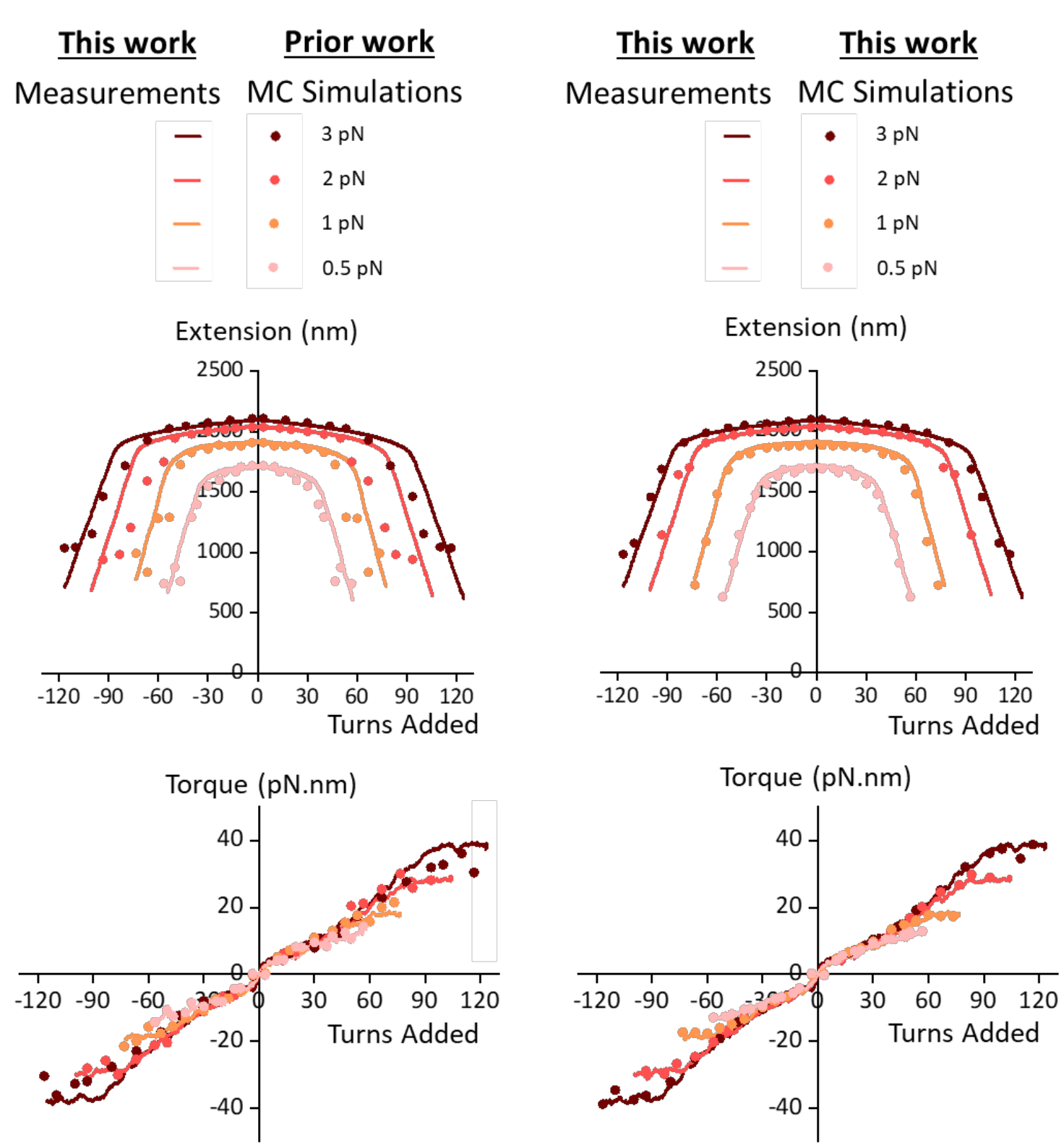

Figure R1. Comparison of braiding mechanics predictions by our MC simulations and by prior MC simulations. To compare these simulations with measurements, we also show the measurements of the 'O substrate' in Figure 2a of this work. To compare with prior work, we reproduced the MC simulations of Charvin et al.[7]. For this preproduction, we used a DNA diameter of 3.5 nm, which yields the best match to the measurements. Nevertheless, the MC simulations of this prior work deviate from measurements as the catenation number increases. In contrast, the MC simulations of this work agree well with the measurements.

References:

1. Jones, M. L., Baris, Y., Taylor, M. R. G. & Yeeles, J. T. P. Structure of a human replisome shows the organisation and interactions of a DNA replication machine. *EMBO J.* **40**, EMBJ2021108819 (2021).
2. Jones, M. L., Aria, V., Baris, Y. & Yeeles, J. T. P. How Pol α-primase is targeted to replisomes to prime eukaryotic DNA replication. *Mol. Cell* **83**, 2911-2924.e16 (2023).
3. Sun, J. *et al.* The architecture of a eukaryotic replisome. *Nat. Struct. Mol. Biol.* **22**, 976–982 (2015).
4. Fernandez-Leiro, R., Conrad, J., Scheres, S. H. & Lamers, M. H. cryo-EM structures of the E. coli replicative DNA polymerase reveal its dynamic interactions with the DNA sliding clamp, exonuclease and τ. *eLife* **4**, e11134 (2015).
5. Gao, Y. *et al.* Structures and operating principles of the replisome. *Science* **363**, eaav7003 (2019).
6. Wang, M. D., Yin, H., Landick, R., Gelles, J. & Block, S. M. Stretching DNA with optical tweezers. *Biophys. J.* **72**, 1335–1346 (1997).
7. Charvin, G., Vologodskii, A., Bensimon, D. & Croquette, V. Braiding DNA: Experiments, Simulations, and Models. *Biophys. J.* **88**, 4124–4136 (2005).
8. Wang, M. D. *et al.* Force and Velocity Measured for Single Molecules of RNA Polymerase. *Science* **282**, 902–907 (1998).
9. Galburt, E. A. *et al.* Backtracking determines the force sensitivity of RNAP II in a factor-dependent manner. *Nature* **446**, 820–3 (2007).
10. Le, T. T. *et al.* Synergistic Coordination of Chromatin Torsional Mechanics and Topoisomerase Activity. *Cell* **179**, 619-631.e15 (2019).
11. Le, T. T. *et al.* Etoposide promotes DNA loop trapping and barrier formation by topoisomerase II. *Nat. Chem. Biol.* **19**, 641–650 (2023).

Response to Reviewers

Re: Nature Communications manuscript NCOMMS-25-06808A-Z
Geometry of Braided DNA Dictates Supercoiling Partitioning by Hong et al.

We greatly appreciate the time and effort of all three reviewers in commenting on our revised manuscript. We found these comments very helpful. Below, we have carefully considered each comment and taken comprehensive actions to address these comments.

Reviewer #1 (Remarks to the Author):

The authors have done a commendable job addressing the reviewers' comments and incorporating substantial revisions into the manuscript. The addition of the refined Monte Carlo simulation framework and the new single-DNA tether experiments strengthen the study, while revisions throughout the text better position the findings within the context of prior work.

Despite these improvements, the study remains primarily a refinement and extension of existing understanding rather than a fundamentally new conceptual advance. As noted by several reviewers, the reported results are largely consistent with expectations arising from previous single-molecule investigations and theoretical models of DNA braiding. Furthermore, the proposed biological significance of the observed torque overshoot remains speculative and is not directly demonstrated.

In my view, this is a rigorous and technically strong study that would be well suited for a specialized journal where the experimental methodology, computational modeling, and quantitative analysis can be more fully appreciated. I agree with the suggestion of journals such as Biophysical Journal or, if the authors wish to remain within the Nature Portfolio, Scientific Reports.

We thank the Reviewer for their careful evaluation of the revised manuscript and for recognizing the rigor of the experimental, computational, and analytical aspects of the study. We appreciate the Reviewer's perspective regarding the degree of conceptual advance. While our work builds on prior investigations of DNA braiding, we believe that the combination of direct torque measurements, defined braiding geometries, and quantitative Monte Carlo modeling provides new mechanistic insight into how supercoiling may be partitioned during replication. We agree that direct *in vivo* validation of this mechanism remains lacking and represents an important direction for future investigation. We are grateful for the Reviewer's positive assessment of the technical quality of the work.

Reviewer #2 (Remarks to the Author):

As I argued in more detail in my previous review, these are novel and important measurements that will be valuable to the field. The study is further strengthened by the inclusion of additional supporting data and by the new MC simulations. There are aspects of the analysis and conclusions that remain unjustified and should be amended or removed — but the work is otherwise appropriate for publication in Nature Communications.

We very much appreciate the positive remarks and detailed comments from Reviewer #2. Upon reading the comment below, we recognized that our previous response did not adequately address the Reviewer's original comments, which were particularly relevant for the analysis used in Supplementary

Fig. 5. We apologize for this oversight and hope that the more detailed and rigorous response provided below adequately addresses the Reviewer's concerns.

To clarify a point: the observed torque overshoot is a property of the equilibrium torque(n) relationship measured in the fixed-n ensemble. In the revised manuscript, this result is further clarified by the inclusion of forward-and-reverse torque(n) curves (Fig. S4) and by MC simulations to probe the equilibrium relationship in this ensemble. If the braid is placed in series with a soft torsional spring such as single DNA duplex (as in Fig. S5), then fluctuations in braid catenation will become accessible, with behavior approaching the fixed-torque ensemble for a sufficiently soft spring — please see my previous comments for additional details. In Fig. S5 and the associated sections of the text, the authors assume without justification that braid catenation in the forked molecule is completely suppressed for torques below the overshoot torque (as defined at fixed-n for the isolated braid). This is now described as a "kinetic barrier" but there is no description or elaboration of a kinetic model or discussion of timescales, and Fig, S5 implies (without explanation) that rates of crossing the barrier are negligible at all relevant timescales and at all torques considered. As the authors note, the geometry in Fig. S5 is in any case fairly contrived for biological contexts — but if they are going to devote space to predicting the behavior of this geometry, it should be better justified or reconsidered. In discussing the overshoot, the authors may also miss an opportunity to relate the phenomenon to previous measurements and models of torque overshoots in DNA structural transitions, including past work from the authors' lab on plectonemic buckling.

We agree with the reviewer that while our measurements were performed for a thermodynamic ensemble with fixed supercoiling, the analysis for Supplementary Fig. 5 requires a thermodynamic ensemble in which the torque in the front of the replisome is balanced by the torque in the back. As the Reviewer clearly noted in their initial comments, this requires a more careful analysis for the condition when there is a torque overshoot. If the torque value rises above the torque gap but below the torque overshoot, the same torque value can be obtained at multiple catenation numbers, because the function of supercoiling versus torque is non-monotonic. In our previous revision, we considered only the smaller catenation number, which would occur when equilibration is slow, and this assumption may not be valid.

In this revision, we followed the Reviewer's initial suggestions by finding the equilibrium solutions. We examined a thermodynamic ensemble of fixed total supercoiling across the forked DNA. The catenation number has a bimodal distribution peaking at a low catenation number before overcoming the torque overshoot (referred to as "pre-hop") and at a high catenation number after overcoming the torque overshoot (referred to as "post-hop"). We find the mean values for any state parameter (such as the catenation number in the braid and the torque of the forked system) using the partition function derived from the bimodal states. This new analysis is now shown in the revised Supplementary Fig. 5. For clarity, we also show the solution obtained by considering only the "pre-hop" state or only the "post-hop" state. As shown in this figure, the "post-hop" and the equilibrium solutions nearly overlap, which is expected since the "post-hop" state has a lower energetic cost. The cartoons above the plots further clarify these states and how supercoiling partitions at each regime of the superhelical density. In the "pre-hop" state, supercoiling only partitions to the front of the replisome, whereas in the "post-hop" state, supercoiling partitions largely to the back of the replisome. We hope this revised SI clearly illustrates both the bimodal distribution and the equilibrium state.

Additional comments: more details should be provided for the MC simulations, including how parameters were chosen, how results were scaled for comparison with experiments, and how torque

curves appear for low catenation number in v-substrates (how closely is the experimental "torque gap" feature recapitulated?). I would also recommend displaying representative chain configurations.

We have followed the Reviewer's suggestion by taking the following actions:

1. We have revised the Methods section related to simulations to clarify how simulation parameters were chosen. In the same section, we have also expanded this section to discuss how simulations were scaled for comparison with experiments on pages 18-20 of the revised manuscript.

2. We have added a new SI figure (Supplementary Fig. 7) to demonstrate that the torsional parameter relations shown in Fig. 4 are consistent when two different DNA contour lengths were used for simulations.

3. We have also added a new SI figure (Supplementary Fig. 8) to show how our simulations clearly show the "torque gap", which is observed experimentally.

4. We have also added a new SI figure (Supplementary Fig. 10) to show representative chain configuration snapshots for O, V, and U substrates. For each substrate, we show two snapshots, one for a pre-buckled state and the other for a post-buckled state.

Reviewer #3 (Remarks to the Author):

I appreciate the significant additional effort the authors put into revising and extending the MC simulations and the additional insights obtained by combining the MC simulation results with the experimental data. The new insights combined with the new tools made available to the community have significantly increased the importance and impact of this work.

The authors have addressed my questions and concerns in their revived manuscript. All together I recommend acceptance.

We greatly appreciate these comments from the Reviewer.

Reviewer #3 (Remarks on code availability):

The code includes a reasonable readme and appears to be complete. It could use some additional comments, but overall the code is sound and it is a useful translation of the original code into matlab, which will make it more user friendly and widely deployable.

We have expanded the readme file to include more comments on the code under the GitHub repository.
